# Inhibition Mechanism of Components Isolated from *Morus alba* Branches on Diabetes and Diabetic Complications via Experimental and Molecular Docking Analyses

**DOI:** 10.3390/antiox11020383

**Published:** 2022-02-14

**Authors:** Ryeong-Ha Kwon, Niha Thaku, Binod Timalsina, Se-Eun Park, Jae-Sue Choi, Hyun-Ah Jung

**Affiliations:** 1 Department of Food Science and Human Nutrition, Jeonbuk National University, Jeonju 54896, Korea; haha8447@gmail.com (R.-H.K.); neehathaku@gmail.com (N.T.); binodtimalsina19@gmail.com (B.T.); 2 Department of Food Science and Nutrition, Pukyong National University, Busan 48513, Korea; gogo1685@naver.com; 3 Department of Biomedical Science, Asan Medical Institute of Convergence Science and Technology, Seoul 05505, Korea

**Keywords:** *Morus alba*, α-glucosidase, protein tyrosine phosphatase 1B, enzyme kinetic, molecular docking analysis, structure–activity relationship

## Abstract

Previously, we reported the anti-diabetic effect of *Morus alba* root bark and the compounds therein. In our continuous study of other parts of this plant, the ability of the branch of *Morus alba* to inhibit α-glucosidase, protein tyrosine phosphatase 1B (PTP1B), and advanced glycation end products (AGEs) formation was evaluated. Moreover, there are no previous studies that have performed enzyme kinetics and molecular docking analyses, along with assessments of peroxynitrite (ONOO^−^) inhibitory activities. Since the *Morus alba* branch exhibited favorable inhibitory effects, repeated column chromatography was performed to obtain eight compounds, including four flavonoids (**1, 3**, **6**, **8**), one arylbenzofuran (**2**), one stilbene (**5**), one Diels–Alder-type adduct (**7**), and one sterol (**4**). Among them, compounds **1**–**3** and **5**–**7** were mixed-type inhibitors of α-glucosidase, sharing the same catalytic residues with acarbose and the same allosteric sites with (Z)-3-bytylidenephthalide. On the other hand, kuwanon C (**1**) and oxyresveratrol (**5**) interacted with residues of the allosteric site (*α*3 and *α*6 helices) of PTP1B, indicating their use as non-competitive inhibitors. Interestingly, kuwanon G (**7**) directly bound the catalytic site, or interrupted the binding between the substrate and the active site, as a mixed-type inhibitor. Moreover, most of the compounds exhibited greater activity against AGE formation and ONOO^−^ than positive controls. The IC_50_ values required to inhibit ONOO^−^ using compounds **1**, **3**, **5**, **6**, and **7** were reported for the first time, and range from 1.08 to 12.92 μM. Based on the structure–activity relationship, the presence of hydroxyl, resorcinol, and prenyl moieties was important in the prevention of diabetes’ pathological mechanisms, and these findings have been further supported by molecular docking analysis. These computational and experimental results will be useful in the development of therapeutic candidates to prevent/treat diabetes and its complications.

## 1. Introduction

Diabetes mellitus (DM) is a chronic metabolic disease characterized by hyperglycemia, which results from carbohydrate metabolic disorders associated with impaired insulin production (type 1) or developed insulin resistance (type 2) [1]. According to the International Diabetes Federation, the worldwide prevalence of DM in 2019 was 463 million, and this is expected to reach 700 million by 2045 [2]. The increased prevalence of DM means that heart attacks, heart failure, blindness, stroke, kidney failure, and depression will also increase, nationally and globally [3].

One therapeutic approach is glucose control, which conclusively induces a decrease in hyperglycemia. Glucose homeostasis is not maintained in diabetic patients, resulting in fasting hypoglycemia, fasting hyperglycemia, and post-prandial hyperglycemia (PPG). In particular, the control of PPG is important in the early treatment and prevention of DM and its complications [4]. In order to treat type 2 DM, metformin is the first-line therapy, but it can cause side effects such as gastrointestinal diseases and anorexia, and some patients also experience a metallic taste [5]. If metformin is unable to reach a suitable glycemic target, additional options are available, one of which is *α*-glucosidase inhibitors [6]. The *α*-glucosidase in the small intestinal membrane catalyzes the hydrolysis of glycosidic bonds in dietary carbohydrates in order to absorb monosaccharides [7]. Its inhibitors can play a crucial role as alternative agents, acting directly to reduce PPG without severe hypoglycemia [4]. Acarbose is an effective *α*-glucosidase inhibitor, but causes side effects, primarily of a gastrointestinal nature, and other disorders in patients who take it for more than 5 years [8]. Alternatively, improvements in insulin sensitivity reduce the abnormal glucose metabolism and cardiovascular risk factors associated with DM. Protein tyrosine phosphatases catalyze protein tyrosine de-phosphorylation and regulate cellular signal transduction and metabolism [9]. In particular, protein tyrosine phosphatase 1B (PTP1B) can dephosphorylate insulin receptors or the insulin receptor substrate, inhibiting insulin signaling and reducing insulin sensitivity.

The prolonged hyperglycemia caused by DM can affect the heart, blood vessels, nerves, eyes, and kidneys, and leads to diabetic complications [10]. Diabetic complications are caused by the advanced glycation end products (AGEs) that are irreversibly produced by the non-enzymatic glycation of proteins. Inhibiting AGE formation prevents diabetic complications, such as diabetic nephropathy, retinopathy, and neuropathy, and reduces cholesterol levels [11]. Aminoguanidine is an AGE formation inhibitor, and it reacts with methylglyoxal, which is an *α*-dicarbonyl compound and produces AGEs [12]. However, it has side effects, such as gastrointestinal disturbance, liver abnormalities, and flu-like symptoms [13]. Moreover, typical reactive oxygen species (ROS) and reactive nitrogen species (RNS) are also associated with DM and its complications, interrupting insulin signaling and reducing insulin sensitivity [14]. Among them, peroxynitrite (ONOO^−^), an RNS, is reported to induce the formation of *N^ε^*-(carboxymethyl)-lysine, a major AGE structure, which is formed by the autoxidation of glucose and the oxidative cleavage of Amadori products [15]. Additionally, this ONOO^−^ is highly related to protein tyrosine nitration, leading to interruptions of tyrosine phosphorylation and insulin signaling. Zhou et al. [16] showed that the upregulation of tyrosine nitration and the downregulation of tyrosine phosphorylation in HepG2 cells was induced by ONOO^−^ in a dose-dependent manner. In addition, ONOO^−^ scavengers may induce increased insulin sensitivity by decreasing nitration and increasing phosphorylation in insulin-resistant mice [17]. Alam et al. [18] reported that plant-derived polyphenols, which have strong antioxidant effects, can significantly delay DM and its complications.

*Morus alba*, the white mulberry, grows widely in many Asian countries, including China, India, Japan, and Korea. Various compounds, including alkaloids, terpenoids, Diels–Alder adducts, stilbenes, phenolic acids, flavonoids (including anthocyanin and chalcones), 2-arylbenzofurans, and coumarins, have been identified in *Morus alba* [19]. *Morus alba* branches are used in traditional Chinese medicine (TCM) and health beverages, and display antioxidant, anti-inflammatory, anti-hyperlipidemia, and anti-diabetic effects [20]. Additionally, *Morus alba* branches have been shown to improve insulin secretion and insulin sensitivity in C57BLKS/J db/db mice [21]. Several studies of *Morus alba* branches have been performed in vivo using extracts, and the compounds isolated from branches have been studied less frequently than those from other parts of the plant (root bark, leaf, and fruit). Therefore, we describe the isolation of bioactive compounds via the EtOAc fraction of *Morus alba* branches, and the evaluation of their antioxidant, anti-diabetic, and anti-diabetic complication-related activities, based on the structure–activity relationship. In addition, the α-glucosidase and PTP1B inhibition mechanisms of the bioactive compounds were evaluated by enzyme kinetic assessment and molecular docking analysis.

## 2. Materials and Methods

### 2.1. General Experimental Procedures

^1^H and ^13^C NMR spectra were collected using a JEOL JNM ECP-400 spectrometer (Tokyo, Japan) at 600, 500, and 400 MHz for ^1^H NMR and 150, 125, and 100 MHz for ^13^C NMR in deuterated solvent (methanol-*d*_4_ (CD_3_OD), dimethyl-sulfoxide-*d*_6_ (CD_3_)_2_SO), and pyridine-*d*_5_ (C_5_D_5_N)). Column chromatography was carried out using Sephadex LH-20 (20–100 µM, Sigma, St. Louis, MO, USA), silica (Si) gel 60 (70–230 mesh, Merck, Darmstadt, Germany), and Lichroprep^®^ RP-18 (40–63 µm, Merck, Darmstadt, Germany). Thin-layer chromatography (TLC) was conducted on pre-coated Merck Kieselgel 60 F_254_ plates (20 × 20 cm, 0.25 mm, Merck) and RP-18 F_254_S plates (5 × 10 cm, Merck) using 10% H_2_SO_4_ (sulfuric acid dissolved in methanol) as a spray reagent. All the solvents used for column chromatography were of reagent grade and obtained from commercial sources.

### 2.2. Chemicals and Reagents

Acarbose, bovine serum albumin, aminoguanidine hydrochloride, diethylene triamine penta-acetic acid (DTPA), dihydrorhodamine 123 (DHR 123), dl-dithiothreitol, d-(−)-fructose, d-(+)-glucose, *p*-nitrophenyl *α*-d-glucopyranoside (*p*NPG), *p*-nitrophenyl phosphate (*p*NPP), ethylenediaminetetraacetic acid, and yeast *α*-glucosidase (from Saccharomyces cerevisiae) were purchased from Sigma-Aldrich Chemical Company (St. Louis, MO, USA). PTP1B was purchased from Enzo Life Sciences (Farmingdale, NY, USA). Peroxynitrite (ONOO^−^) and sodium azide were purchased from Bio Rad Laboratories Calbiochem (San Diego, CA, USA) and JUNSEI (Chuo-ku, Tokyo, Japan), respectively.

### 2.3. Plant Material

The branches of *Morus alba* were collected from Yeongcheon, Andong, and Jecheon Provinces, Republic of Korea, and were purchased at Kyungdongmart, Seoul, Korea, in July 2017. A voucher specimen of the branches was registered and deposited at the Department of Food Science and Human Nutrition, Jeonbuk National University, Jeonju, South Korea (Professor Jung, H.A.).

### 2.4. Extraction, Fractionation, and Isolation

**Figure 1:**
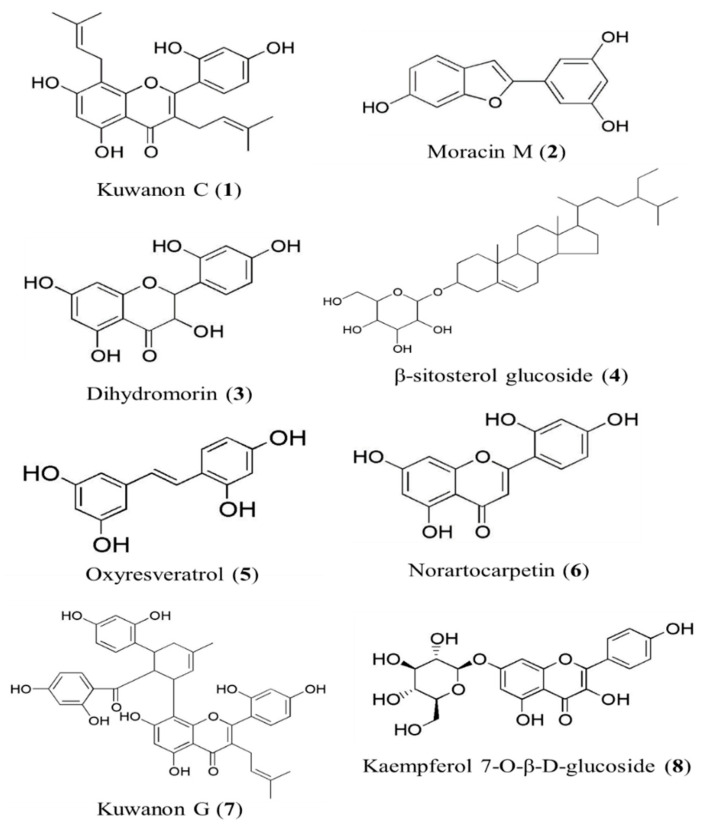
The structure of compounds (**1**–**8**) isolated from *Morua alba* branches.

Dried branches of *Morus alba* (7.2 kg) were extracted using hot methanol (MeOH) and reflux extraction (6 L × 5 times). After filtration using a funnel, the total filtrate was concentrated on a rotary evaporator at 80 °C to acquire the MeOH extract (789.31 g). This extract was then suspended in distilled water (H_2_O) and successively partitioned with methylene chloride (CH_2_Cl_2_), ethyl acetate (EtOAc), and n-butanol (n-BuOH), and the H_2_O residue was also retained (Scheme S1). The EtOAc (66.82 g) fraction (MB-EF) was first chromatographed on a Si gel column with a mixed solvent of CH_2_Cl_2_, MeOH, and H_2_O (50:1:0 to 5:1:0.1, gradient solvent conditions) to obtain 26 subfractions (MB-EF-CM1–26). MB-EF-CM10 (3.61 g) was chromatographed on Sephadex LH-20 with MeOH, yielding 13 subfractions (MB-EF-CM10-Sep1–13). After TLC, MB-EF-CM10-Sep5 (1.9 g) was subjected to column chromatography using Si gel with a solvent mixture of *n*-hexane:EtOAc:MeOH (5:2:0.1 to 0:1:1, gradient conditions) to obtain kuwanon C (**1**, 30 mg) [22]. MB-EF-CM13 was chromatographed on Sephadex LH-20 with MeOH, yielding 10 subfractions (MB-EF-CM13-Sep1–10). Fraction 7 (233.2 mg) of MB-EF-CM13 was chromatographed on Si gel with a solvent mixture of CH_2_Cl_2_:MeOH (20:1 to 1:1, gradient conditions), yielding 5 subfractions (MB-EF-CM13-Sep7-CM1–5). Fraction 2 (104.9 mg) of MB-EF-CM13-Sep7 was chromatographed on Si gel with a solvent mixture of *n*-hexane:EtOAc:MeOH (5:3:0.1, gradient condition) and medium-pressure liquid chromatography (MPLC) with aqueous acetonitrile (ACN) (1:9, gradient condition) to obtain moracin M (**2**, 40 mg) [23]. Moracin M (**2**, 31 mg) was also isolated from fractions (11 + 12) of MB-EF by column chromatography on Sephadex LH-20 with MeOH and Si gel using a solvent mixture of *n*-hexane:EtOAc:MeOH (5:3:0.1, gradient condition) and MPLC with aqueous MeOH (3:7, gradient condition). Dihydromorin (**3**, 41.3 mg) [24] was isolated from fraction 14 of MB-EF by column chromatography on Sephadex LH-20 with MeOH and Si gel using a solvent mixture of CH_2_Cl_2_:MeOH (20:1, gradient condition) and MPLC with aqueous MeOH (1:4, gradient condition). MB-EF-CM14 was chromatographed on Sephadex LH-20 and recrystallized with MeOH to obtain *β*-sitosterol glucoside (**4**, 26.7 mg) [25]. Fraction 4 from MB-EF-14-(6–9) was subjected to MPLC with aqueous MeOH (1:5, gradient condition) to isolate oxyresveratrol (**5**, 1.5 g) [26], and purification was conducted to obtain norartocarpetin (**6**, 8.5 mg) [27]. Norartocarpetin (**6**, 25.1 mg) was also isolated from fraction 13 of MB-EF by column chromatography on Sephadex LH-20, Si gel, and MPLC with aqueous MeOH (2:3, gradient condition). MB-EF-CM(15 + 16) was chromatographed on Sephadex with MeOH, yielding 7 subfractions (MB-EF-CM(15 + 16)-Sep1–7). Fraction 5 of MB-EF-CM(15 + 16) was again chromatographed on Si gel using a solvent mixture of CH_2_Cl_2_:MeOH (30:1, gradient condition) and subjected to column chromatography using MPLC with aqueous ACN (0:1, gradient condition) to isolate kuwanon G (**7**, 38.4 mg) [28]. MB-EF-CM(20 + 21) was chromatographed on Si gel with EtOAc:MeOH (250:1, gradient condition), yielding 10 subfractions (MB-EF-CM(20 + 21)-EM1–10). Fraction 6 of MB-EF-CM(20 + 21) was chromatographed using MPLC with aqueous ACN (1:4, gradient condition), and subfraction 7 was recrystallized with MeOH to obtain kaempferol 7-*O*-*β*-d-glucopyranoside (**8**, 12.6 mg). MB-EF-CM22 was chromatographed on Si gel with EtOAc:MeOH (200:1, gradient condition), yielding 10 subfractions (MB-EF-CM22-EM1–10). Fraction 6 of MB-EF-CM22 was chromatographed on Si gel with CH_2_Cl_2_:MeOH:H_2_O (40:10:0.1, gradient condition) and MPLC with aqueous ACN (1:4, gradient condition) and was recrystallized with MeOH, which also yielded kaempferol 7-*O*-*β*-d-glucopyranoside (**8**, 7.6 mg) [29] (Scheme S2). All compounds were characterized and identified by spectroscopic analyses (^1^H-NMR and ^13^C-NMR) and compared with published data (Tables S1 and S2). The structures of these compounds are shown in Figure 1.

#### 2.4.1. Kuwanon C (**1**)

^1^H NMR (CD_3_OD, 400 MHz) *δ* 7.08 (1H, d, *J* = 8.4 Hz, H-6′), 6.44 (1H, d, *J* = 2.0 Hz, H-3′), 6.41 (1H, dd, *J* = 2.4, 8.4 Hz, H-5′), 6.24 (1H, s, H-6), 5.17 (1H, t, *J* = 6.0 Hz, H-2′′′), 5.10 (1H, t, *J* = 5.2 Hz, H-2′′), 3.36 (2H, s, H-1′′′), 3.11 (2H, d, *J* = 6.8 Hz, H-1′′), 1.60 (3H, s, H-5′′′), 1.58 (3H, s, H-4′′′), 1.56 (3H, s, H-5′′), 1.40 (3H, s, H-4′′); ^13^C NMR: (CD_3_OD, 100 MHz) *δ* 184.0 (C-4), 163.6 (C-7), 162.6 (C-8a), 161.7 (C-2), 160.6 (C-4′), 157.8 (C-2′), 157.0 (C-5), 132.6 (C-3′′), 132.4 (C-3′′), 132.0 (C-6′) 123.4 (C-2′′), 122.9 (C-2′′), 121.3 (C-3), 113.5 (C-1′), 107.8 (C-5′), 107.5 (C-8), 105.3 (C-4a), 103.7 (C-3′), 98.9 (C-6), 25.9 (C-5′′′), 25.8 (C-5′′), 24.8 (C-1′′), 22.3 (C-1′′), 17.7 (C-4′′), 17.6 (C-4′′).

#### 2.4.2. Moracin M (**2**)

^1^H NMR (CD_3_OD, 500 MHz), *δ* 7.35 (1H, d, *J* = 8.0 Hz, H-4), 6.92 (1H, s, H-3), 6.92 (1H, d, *J* = 1.5 Hz, H-7), 6.78 (1H, d, *J* = 2.0 Hz, H-2′), 6.78 (1H, d, *J* = 2 Hz, H-6′), 6.75 (1H, dd, *J* = 2.0, 8.0 Hz, H-5), 6.27 (1H, t, *J* = 2.5 Hz, H-4′); ^13^C NMR (CD_3_OD, 125 MHz) *δ* 159.9 (C-3′, 5′), 157.2 (C-7a), 156.8 (C-6), 156.1 (C-2), 133.8 (C-1′), 123.0 (C-3a), 122.0 (C-4), 113.3 (C-5), 103.9 (C-2′, 6′), 103.5 (C-4′), 102.2 (C-3), 98.4 (C-7).

#### 2.4.3. Dihydromorin (**3**)

^1^H NMR: (CD_3_OD, 500 MHz) *δ* 7.22 (1H, d, *J* = 9.0 Hz, H-6′), 6.37 (1H, d, *J* = 5.4 Hz, H-3′), 6.36 (1H, d, *J* = 1.2 Hz, H-5′), 5.92 (1H, d, *J* = 1.8 Hz, H-8), 5.88 (1H, d, *J* = 1.2 Hz, H-6), 5.39 (1H, d, *J* = 12 Hz, H-2), 4.79 (1H, d, *J* = 12 Hz, H-3); ^13^C NMR (CD_3_OD, 125 MHz) *δ* 198.9 (C-4), 168.5 (C-5), 165.2 (C-8a), 164.9 (C-7), 160.1 (C-4′), 158.5 (C-2′), 130.9 (C-6′), 115.5 (C-1′), 107.9 (C-5′), 103.6 (C-3′), 101.8 (C-4a), 97.1 (C-8), 96.2 (C-6), 79.9 (C-2), 72.4 (C-3).

#### 2.4.4. β-Sitosterol Glucoside (**4**)

^1^H NMR (C_5_D_5_N, 600 MHz) *δ* 5.34 (1H, d, *J* = 4.8 Hz, H-6), 5.04 (1H, d, *J* = 7.2 Hz, H-1′), 1.06 (3H, m, H-21), 0.98 (3H, d, *J* = 6.6 Hz, H-19), 0.92 (3H, s, H-26), 0.88 (3H, t, *J* = 6.0 Hz, H-29), 0.86 (3H, t, *J* = 6.0 Hz, H-27), 0.65 (3H, s, H-18); ^13^C NMR (C_5_D_5_N, 150 MHz) *δ* 140.9 (C-5), 121.9 (C-6), 102.6 (C-1′), 78.6 (C-3), 78.5 (C-3′), 78.1 (C-5ʹ), 75.3 (C-2′), 71.7 (C-4′), 62.8 (C-6′), 56.8 (C-14), 56.2 (C-17), 50.3 (C-9), 46.0 (C-24), 42.5 (C-13), 40.0 (C-4), 39.3 (C-12), 37.5 (C-1), 36.9 (C-10), 36.4 (C-20), 34.2 (C-22), 32.2 (C-7), 32.1 (C-8), 30.3 (C-2), 29.5 (C-25), 28.6 (C-16), 26.4 (C-23), 24.5 (C-15), 23.4 (C-28), 21.3 (C-11), 20.0 (C-27), 19.4 (C-19), 19.2 (C-26), 19.0 (C-21), 12.2 (C-29), 12.0 (C-18).

#### 2.4.5. Oxyresveratrol (**5**)

^1^H NMR (CD_3_OD, 400 MHz) *δ* 7.32 (1H, d, *J* = 6.0 Hz, H-6), 7.26 (1H, d, *J* = 16 Hz, H-*β*), 6.80 (1H, d, *J* = 16.4 Hz, H-*α*), 6.43 (2H, d, *J* = 2.4 Hz, H-2′, 6′), 6.30 (2H, m, H-3, 5), 6.12 (1H, t, *J* = 2.4 Hz, H-4′); ^13^C NMR (CD_3_OD, 100 MHz) *δ* 159.6 (C-3′, 5′), 159.2 (C-4), 157.3 (C-2), 142.2 (C-1′), 128.4 (C-6), 126.5 (C-*β*), 124.8 (C-*α*), 117.8 (C-1), 108.3 (C-3), 105.6 (C-2′, 6′), 103.5 (C-5), 101.2 (C-4′).

#### 2.4.6. Norartocarpetin (**6**)

^1^H NMR (CD_3_OD, 500 MHz) *δ* 7.77 (1H, d, *J* = 8.5 Hz, H-6′), 7.14 (1H, s, H-3), 6.46 (1H, dd, *J* = 2.4, 8.5 Hz, H-5′), 6.42 (1H, d, *J* = 3.0 Hz, H-3′), 6.42 (1H, d, *J* = 1.8 Hz, H-8), 6.19 (1H, d, *J* = 2.5 Hz, H-6); ^13^C NMR (CD_3_OD, 125 MHz) *δ* 184.3 (C-4), 165.9 (C-2), 164.1 (C-7), 163.3 (C-8a), 163.0 (C-5), 160.4 (C-4′), 159.4 (C-2′), 131.0 (C-6′), 110.7 (C-1′), 109.1 (C-5′), 108.2 (C-3), 105.1 (C-4a), 104.1 (C-3′), 99.8 (C-6), 94.8 (C-8).

#### 2.4.7. Kuwanon G (**7**)

^1^H NMR (CD_3_OD, 600 MHz) *δ* 7.34 (1H, brs, H-27 or H-6′), 7.15 (1H, d, *J* = 8.4 Hz, H-27 or H-6′), 6.75 (1H, d, *J* = 7.2 Hz, H-33), 6.50 (1H, s, H-5′), 6.47 (1H, brs, H-3′), 6.14 (1H, brs, H-30), 6.08 (1H, dd, *J* = 2.4, 8.4 Hz, H-32), 5.94 (2H, s, H-6, 24), 5.90 (1H, d, *J* = 8.4 Hz, H-26), 5.19 (1H, brs, H-15), 5.17 (1H, t, *J* = 7.2 Hz, H-10), 4.58 (1H, brd, *J* = 33 Hz, H-20), 4.34 (1H, d, *J* = 8.4 Hz, H-14), 3.35 (1H, s, H-19), 3.19 (2H, br, H-9), 1.95 (2H, br, H-18), 1.64 (3H, s, H-12), 1.49 (3H, brs, H-17), 1.46 (3H, s, H-13); ^13^C NMR (CD_3_OD, 125 MHz) *δ* 210.2 (C-21), 183.9 (C-4), 165.9 (C-23), 165.7 (C-25, 7), 162.5 (C-2, 8a, 4′), 161.8 (C-29, 2′), 161.1 (C-31), 157.8 (C-5), 134.4 (C-16, 33), 132.7 (C-11, 27, 6′), 124.6 (C-15), 123.0 (C-10, 28), 121.7 (C-3), 115.9 (C-22), 113.8 (C-1′), 108.6 (C-8, 26), 108.2 (C-32), 108.0 (C-5′), 105.7 (C-4a), 103.7 (C-24), 103.6 (C-3′), 102.9 (C-30), 98.5 (C-6), 49.9 (C-20), 39.1 (C-18), 25.9 (C-12, 17), 24.7 (C-9), 23.1 (C-14, 19), 17.7 (C-13).

#### 2.4.8. Kaempferol 7-*O*-β-d-glucopyranoside (**8**)

^1^H NMR ((CD_3_)_2_SO, 500 MHz) *δ* 12.4 (OH, s, OH-5), 8.06 (2H, d, *J* = 8.5 Hz, H-2′, 6′), 6.93 (2H, d, *J* = 9 Hz, H-3′, 5′), 6.79 (1H, d, *J* = 2.5 Hz, H-8), 6.41 (1H, d, *J* = 1.5 Hz, H-6), 5.05 (1H, d, *J* = 7 Hz, H-1′′); ^13^C NMR (C_2_D_6_OS, 125 MHz) *δ* 176.1 (C-4), 162.7 (C-7), 160.3 (C-5), 159.4 (C-4′), 155.7 (C-8a), 147.5 (C-2), 136.0 (C-3), 129.6 (C-2′, 6′), 121.5 (C-1′), 115.5 (C-3′, 5′), 104.7 (C-4a), 99.9 (C-1′′), 98.8 (C-6), 94.4 (C-8), 77.1 (C-5′), 76.4 (C-3′′), 73.1 (C-2′′), 69.6 (C-4′′), 60.6 (C-6′′).

### 2.5. In Vitro Assay for α-Glucosidase Inhibitory Activity

This enzyme inhibition study was carried out spectrophotometrically using a previously published procedure [30]. The α-glucosidase enzyme (0.1 unit) was added to a plate with or without sample dissolved in 10% DMSO with 2.5 mM *p*NPG. The plate was incubated at 37 °C for 10 min, and 0.2 M sodium carbonate solution was added to stop the reaction. The absorbance was measured immediately at 405 nm using a microplate spectrophotometer (Molecular Devices., San Jose, CA, USA). In the control, the sample solution was replaced with the same volume of phosphate buffer in the same volume of reaction mixture. Acarbose was dissolved in 10% DMSO and used as the positive control.

### 2.6. In Vitro Assay for PTP1B Inhibitory Activity

This enzyme inhibition study was carried out spectrophotometrically using a previously published procedure [31]. Recombinant PTP1B enzyme (0.5 unit) was added to a plate with or without sample dissolved in 10% DMSO. After the plate was pre-incubated at 37 °C for 10 min, the substrate (2 mM *p*NPP) was added. Following incubation at 37 °C for 20 min, the reaction was terminated with the addition of 10 M NaOH. The amount of *p*-nitrophenyl produced from the *p*NPP after enzymatic dephosphorylation was estimated by measuring absorbance at 405 nm using a microplate spectrophotometer (Molecular Devices). In the control, the sample solution was replaced by the same volume of phosphate buffer in the same volume of reaction mixture. Ursolic acid was used as the positive control.

### 2.7. In Vitro Assay for Inhibition of AGE Formation

The inhibition of AGE formation was evaluated using a modification of a previously published method [32]. To prepare the reaction solution, 10 mg/mL of bovine serum albumin was added to 0.2 M fructose, 0.2 M glucose, and 3 M sodium azide. The reaction solution was mixed with various concentrations of the samples dissolved in 10% DMSO until the total volume was 1 mL. After an 8-day incubation at 37 °C, the fluorescence intensity of the reaction products was measured using a VERSA MAX GEMINI XPS fluorescence microplate reader (Molecular Devices) with excitation and emission wavelengths of 350 nm and 450 nm, respectively. After obtaining the results, we determined the IC_50_ values as the result on day 4. Aminoguanidine hydrochloride was used as the positive control.

### 2.8. In Vitro Assay for Peroxynitrite Scavenging Activity

The ONOO^−^ scavenging activity assay was conducted using a previously published method [33] that involved measuring highly fluorescent rhodamine 123, which is rapidly converted from non-fluorescent DHR 123 in the presence of ONOO^−^. Briefly, the rhodamine buffer (pH 7.4) was composed of 100 μM DTPA and 5 μM DHR 123. The assay buffer was prepared prior to use and placed on ice. The fluorescence intensity of the oxidized DHR 123 in the background and sample was measured 30 s after treatment with (and without) 200 μM authentic ONOO^−^. The fluorescence intensity of the oxidized DHR 123 was measured using a VERSA MAX GEMINI XPS fluorescence microplate reader (Molecular Devices., CA, USA) at excitation and emission wavelengths of 485 and 530 nm, respectively. l-Penicillamine was used as the positive control.

### 2.9. Kinetic Study for α-Glucosidase and PTP1B Inhibition

Two kinetic methods, using Lineweaver–Burk plots and Dixon plots, were employed to determine the inhibition mechanism [34,35,36]. To obtain the inhibition constant (*K*_i_) of each compound, Dixon plots were calculated by monitoring the effects of various concentrations of the substrates (0.625, 1.25, and 2.5 mM *p*NPG for α-glucosidase and 0.4, 0.5, 1.6, and 3.2 mM *p*NPP for PTP1B). Lineweaver–Burk plots for inhibition of α-glucosidase were obtained using different concentrations of test compounds (14.79, 5.92, 2.35, and 0 µM for kuwanon C (**1**); 20.64, 10.32, 4.13, and 0 µM for moracin M (**2**); 82.17, 41.08, 8.22, and 0 µM for dihydromorin (**3**); 1.02, 0.41, and 0 µM for oxyresveratrol (**5**); 43.67, 8.73, 3.19, and 0 µM for norartocarpetin (**6**); and 1.44, 0.72, 0.38, and 0 µM for kuwanon G (**7**)). Similarly, in tests for PTP1B inhibition, the concentrations of the test compounds were 118.3, 47.3, 23.7, and 0 µM for kuwanon C (**1**); 2, 1, and 0 µM for oxyresveratrol (**5**); and 14.4, 7.2, and 0 µM for kuwanon G (**7**). The enzymatic procedure followed the aforementioned assay method.

### 2.10. Molecular Docking Analysis for α-Glucosidase and PTP1B Inhibition

To investigate the protein–ligand interactions at the molecular level, molecular docking analyses were conducted with compounds **1**–**3** and **5**–**7** using AutoDock 4.2. The structure of α-glucosidase and its catalytic ligand *α*-d-glucose (PDB ID: 3A4A) (with a resolution of 1.6 Å [37]) and the structures of acarbose, a catalytic inhibitor, and (Z)-3-bytylidenephthalide (BIP), an allosteric inhibitor, were obtained from the RCSB Protein Data Bank website. The complex structure of PTP1B with a selective catalytic inhibitor, 3-({5-[*N*-acetyl-3-{4-[(carboxycarbonyl)(2-carboxyphenyl)amino]-1-naphthyl}-l-alanyl)amino]pentyl}oxy)-2-naphthoic acid (compound A) (PDB ID: 1NNY), with a resolution of 2.4 Å [38], and a selective allosteric inhibitor, 3-(3,5-dibromo-4-hydroxy-benzoyl)-2-ethyl-benzofuran-6-sulfonic acid (4-sulfamoyl-phenyl)-amide (compound B) (PDB ID: 1T49), with a resolution of 1.9 Å [39], were obtained from the RCSB Protein Data Bank website. The compounds were docked into the binding sites of the enzyme, and the residues were defined as 5–6 Å in the original complex. The lowest interaction energy between the inhibitors and the enzyme was demonstrated using hydrogen bonds (H-bonds), hydrophobic interactions, and electrostatic interactions. The binding areas of acarbose, BIP, compound A, and compound B were regarded as the most suitable regions for ligand binding in the docking analysis.

## 3. Results

### 3.1. Inhibitory Activity of the Methanol Extract and Its Fractions on α-Glucosidase, PTP1B, AGEs, and Peroxynitrite

To investigate the anti-diabetic components of *Morus alba* branch extract, the MeOH extract and its fractions were tested in inhibition assays. The EtOAc fraction showed a significantly greater inhibitory activity against *α*-glucosidase and AGEs than the positive controls, with IC_50_ values of 2.74 ± 0.15 and 6.40 ± 0.31 μg/mL, respectively. This fraction showed moderate inhibitory activity against PTP1B and ONOO^−^ compared with the positive controls, with IC_50_ values of 8.09 ± 0.08 and 6.74 ± 0.15 μg/mL, respectively. This fraction showed more promising activity than the MeOH extract and other fractions (Table 1).

### 3.2. Inhibitory Activity of Isolated Compounds on α-Glucosidase and PTP1B

Compounds **1**–**3** and **5**–**7** isolated from the EtOAc fraction were examined using *α*-glucosidase and PTP1B inhibition assays. The inhibitory activities of the test compounds against those two enzymes are shown in Table 2. Most compounds showed significantly greater inhibitory activity toward *α*-glucosidase than acarbose, with an IC_50_ value of 350.9 ± 17.94 μM. Among the test compounds, kuwanon G (**7**) exhibited the most potent activity against *α*-glucosidase, with an IC_50_ value of 1.44 ± 0.11 μM, followed by oxyresveratrol (**5**), kuwanon C (**1**), norartocarpetin (**6**), moracin M (**2**), and dihydromorin (**3**). The PTP1B inhibition results showed a similar tendency. Oxyresveratrol (**5**) showed the most potent activity against PTP1B, with an IC_50_ value of 2.85 ± 0.30 μM, followed by kuwanon G (**7**), kuwanon C (**1**), dihydromorin (**3**), and moracin M (**2**). Moracin M (**2**) and dihydromorin (**3**) showed weaker inhibitory activities toward PTP1B than ursolic acid, and norartocarpetin (**6**) showed no PTP1B-inhibitory activity at 100 μg/mL.

### 3.3. Inhibitory Activity of Isolated Compounds against AGE Formation and Peroxynitrite

Compounds **1**–**3** and **5**–**7** isolated from the EtOAc fraction were examined using AGE formation inhibition and ONOO^−^ scavenging assays. As shown in Table 2, most of the tested compounds showed stronger activities than the positive controls. Moracin M (**2**) exhibited the most potent activity against AGE formation and ONOO^−^, with IC_50_ values of 2.10 ± 0.22 μM and 1.08 ± 0.04 μM, respectively. On the other hand, kuwanon C (**1**) showed moderate scavenging activity in the ONOO^−^ assay, but no inhibitory activity on AGE formation at 100 μg/mL.

**Table 2:** *α*-glucosidase, PTP1B, AGE formation, and peroxynitrite inhibitory activities of compounds isolated from *Morus alba* branches.

Compounds	α-Glucosidase			PTP1B			AGEs	ONOO^−^
	IC_50_ (μM) ^1^	Inhibition Mode ^2^	*K*_i_ (μM) ^3^	IC_50_ (μM) ^1^	Inhibition Mode ^2^	*K*_i_ (μM) ^3^	IC_50_ (μM) ^1^	
Kuwanon C (**1**)	14.75 ± 0.88	Mixed	6.85	41.43 ± 1.64	Non-competitive	39.43	>100 ^b^	12.92 ± 0.68
Moracin M (**2**)	32.43 ± 1.65	Mixed	3.32	333.1 ± 20.53	-	-	2.10 ± 0.22	1.08 ± 0.04
Dihydromorin (**3**)	47.35 ± 2.25	Mixed	10.22	180.2 ± 0.77	-	-	117.5 ± 7.89	2.26 ± 0.12
Oxyresveratrol (**5**)	1.86 ± 0.20	Mixed	1.14	2.85 ± 0.30	Non-competitive	2.16	7.56 ± 0.15	2.37 ± 0.21
Norartocarpetin (**6**)	31.95 ± 1.72	Mixed	19.90	>100 ^5^	-	-	77.29 ± 9.58	3.01 ± 0.15
Kuwanon G (**7**)	1.44 ± 0.11	Mixed	2.03	16.17 ± 0.29	Mixed	12.41	69.07 ± 1.49	6.35 ± 0.36
Acarbose ^4^	350.9 ± 17.94	-	-	-	-	-	-	-
Ursolic acid ^4^	-	-	-	16.48 ± 2.07	-	-	-	-
Aminoguanidine ^4^	-	-					890.3 ± 70.16	-
l-Penicillamine ^4^	-	-					-	6.69 ± 0.52

^1^ The 50% inhibition concentration (IC_50_) is expressed as the mean ± SD of triplicate experiments. ^2^ Inhibition type was determined using Lineweaver–Burk plots. ^3^ Inhibition constant (*K*_i_) was determined using Dixon plots. ^4^ Positive controls used in the assays. ^5^ Not determined at 100 μg/mL.

### 3.4. Enzyme Kinetic Study of Compounds in α-Glucosidase and PTP1B Inhibition

**Figure 2:**
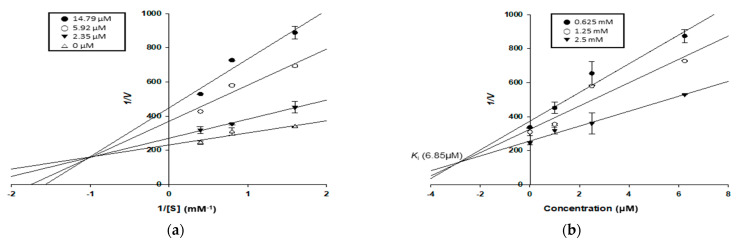

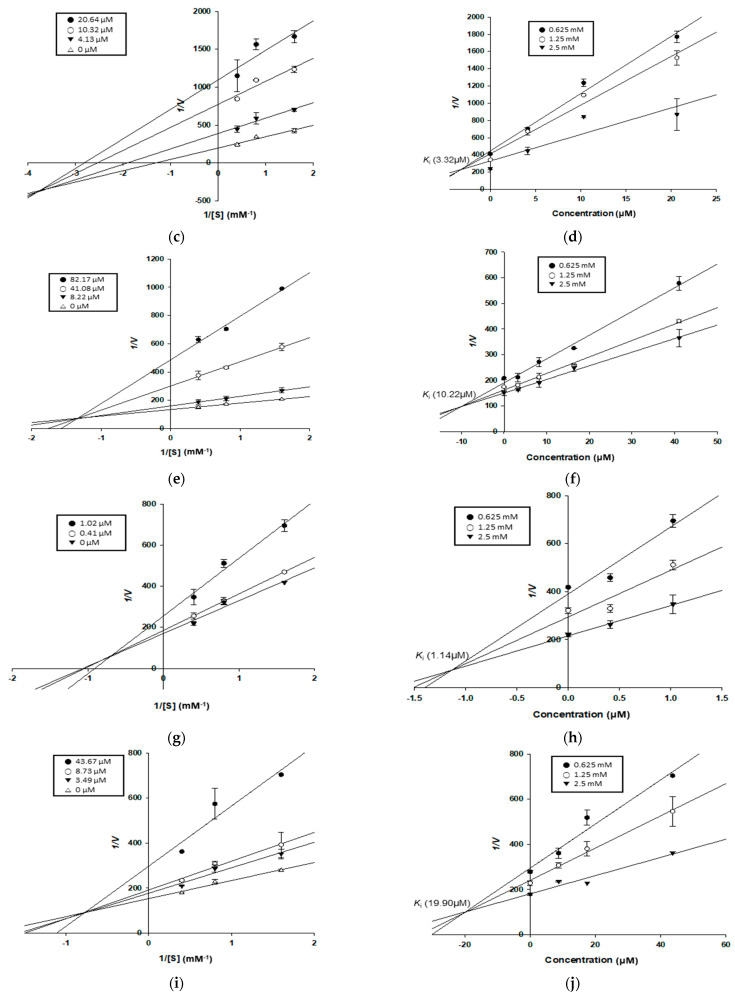

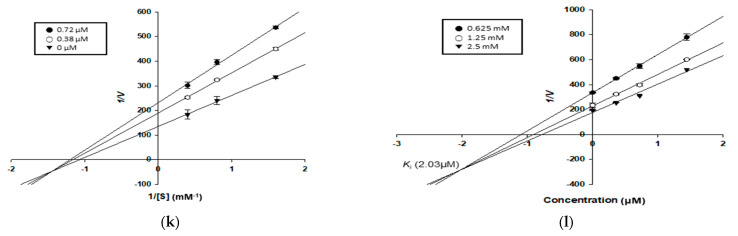
Lineweaver–Burk plots (**a**,**c**,**e**,**g**,**i**,**k**) and Dixon plots (**b**,**d**,**f**,**h**,**j**,**l**) of α-glucosidase inhibition by compounds **1**–**3** and **5**–**7**, respectively: (**a,b**) for kuwanon C (**1**); (**c,d**) for moracin M (**2**); (**e,f**) for dihydromorin (**3**); (**g,h**) for oxyresveratrol (**5**); (**i,j**) for norartocarpetin (**6**); (**k,l**) for kuwanon G (**7**).

**Figure 3:**
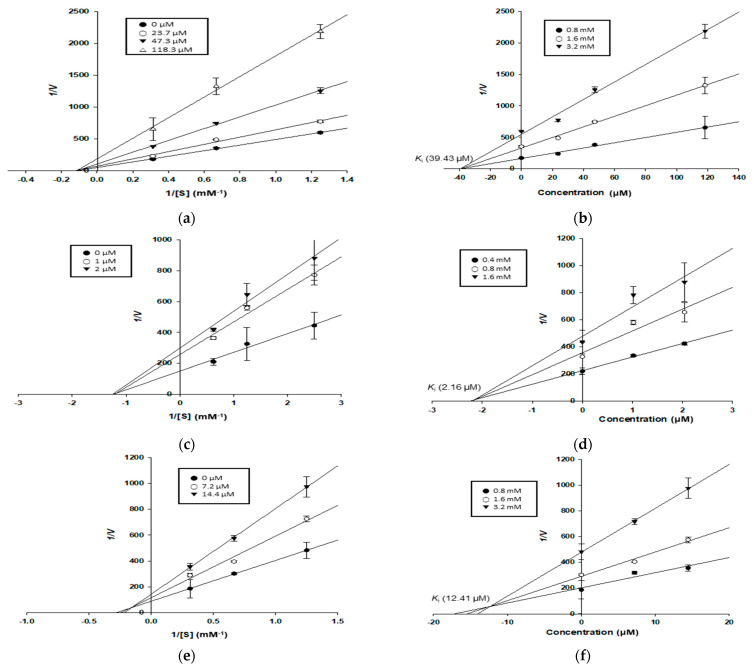
Lineweaver–Burk plots (**a**,**c**,**e**) and Dixon plots (**b**,**d**,**f**) of PTP1B inhibition by compounds **1**, **5**, and **7**, respectively: (**a**,**b**) for kuwanon C (**1**); (**c**,**d**) for oxyresveratrol (**5**); (**e**,**f**) for kuwanon G (**7**).

To explain the type of *α*-glucosidase and PTP1B inhibition demonstrated by the test compounds, enzyme kinetic analyses were conducted at various concentrations of *p*NPG and *p*NPP, respectively. Each line of inhibitors intersected on the *xy*-side of the Lineweaver–Burk plots, indicating mixed-type inhibition. On the other hand, each line of inhibitors also intersected at the same point plots on the *x*-axis, indicating non-competitive inhibition [36]. In Dixon plots, the *K*_i_ value for an enzyme–inhibitor complex is determined via the value of the *x*-axis, which implies −*K*_i_ [35]. All test compounds (**1**–**3** and **5**–**7**) appeared to be mixed-type inhibitors for the α-glucosidase enzyme, with *K*_i_ values of 6.85, 3.32, 10.22, 1.14, 19.90, and 2.03 μM, respectively. Kuwanon (**7**) also exhibited a mixed-type inhibition mode for PTP1B, with a *K*_i_ value of 12.41 μM. In contrast, kuwanon C (**1**) and oxyresveratrol (**5**) appeared to be non-competitive inhibitors for the PTP1B enzyme, with *K*_i_ values of 39.43 and 2.16 μM, respectively (Table 2, Figure 2 and Figure 3).

### 3.5. Molecular Docking Analysis for α-Glucosidase Inhibition

**Figure 4:**
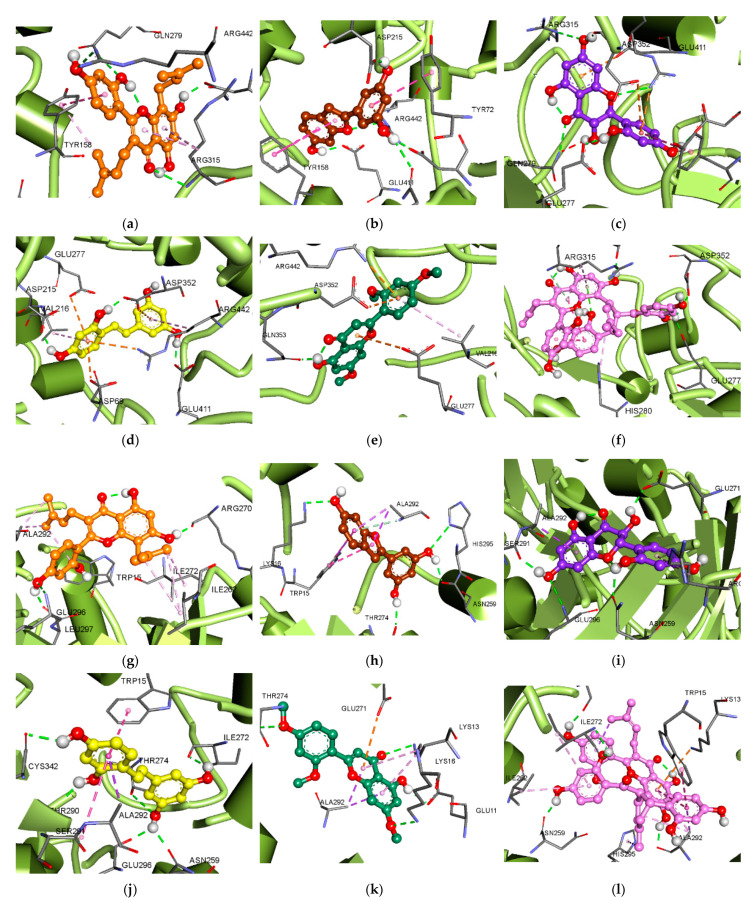
Molecular docking models of α-glucosidase inhibition at catalytic (**a**–**f**) and allosteric sites (**g**–**l**) by compounds **1**–**3** and **5**–**7**, respectively: (**a**,**g**) for kuwanon C (**1**); (**b**,**h**) for moracin M (**2**); (**c,i**) for dihydromorin (**3**); (**d**,**j**) for oxyresveratrol (**5**); (**e**,**k**) for norartocarpetin (**6**); (**f**,**l**) for kuwanon G (**7**).

Molecular docking analyses for compounds **1**–**3** and **5**–**7** with α-glucosidase were conducted. The ligand–enzyme complexes of the six test compounds and acarbose and BIP were rigidly placed in the same pocket of α-glucosidase using AutoDock 4.2. The binding energies of the test compounds, along with their H-bond, hydrophobic, and electrostatic interactions (as shown by the green, pink, and orange lines, respectively), are listed in Table 3, Figure 4 and Figure S1. The kuwanon C (**1**)–*α*-glucosidase complex at the catalytic site presented a −6.66 kcal/mol binding energy, with four H-bonds formed via interactions between three hydroxyl (OH) groups and one carbonyl group and the Gln279, Arg315, Arg442, and Asp307 residues. In addition, kuwanon C (**1**) showed the same hydrophobic interacting residues, Tyr158 and Phe303, as acarbose, the catalytic inhibitor (Figure 4a). In the allosteric inhibition mode, five H-bonds were formed between the four OH groups of kuwanon C (**1**) and the Glu296, Ser298, Leu297, Glu271, and Arg270 residues. The alkyl groups and aromatic ring structure exhibited hydrophobic interactions with Ala292, Lys13, Ile263, Ile272, Ile262, and Arg263 residues, and a π–anion interaction with the Glu271 residue, respectively, sharing Ala292 and Lys13 residues in the allosteric site with BIP (Figure 4g). Catalytic inhibition by moracin M (**2**) gave rise to five H-bonds between three OH groups and oxygen in the benzofuran structure and the Arg442, Asp69, Gln182, Asp215, and Glu411 residues (−7.73 kcal/mol). Moracin M (**2**) also exhibited a hydrophobic interaction with the Tyr72 and Tyr158 residues, and showed a π–cation interaction between the aromatic ring and the Arg442 residue (Figure 4b). The moracin M (**2**)–*α*-glucosidase complex at the allosteric site displayed four H-bonds between the 6-, 3′-, and 5′-OH groups and the Lys16, His295, Asn259, and Thr274 residues, and exhibited hydrophobic interactions with Trp15 and Ala292 residues (Figure 4h). In the catalytic inhibition mode, six H-bonds formed between three OH groups and a carbonyl group of dihydromorin (**3**) and the Gln279, Arg315, Arg442, Asp69, Glu277, and Asp352 residues. Dihydromorin (**3**) also interacted with the Tyr72 residue, via hydrophobic interaction, and showed a π–cation (Arg442) and two π–anion (Asp352 and Glu411) interactions (Figure 4c). On the other hand, the binding energy to the allosteric site was −6.93 kcal/mol, forming four H-bonds between three OH groups and the Glu296, Asn259, Glu271, and Ser291 residues. Dihydromorin (**3**) also showed hydrophobic interactions with Ala292 and Arg263 residues (Figure 4i). Oxyresveratrol (**5**) showed the same amino acid residues as acarbose, such as Asp352 (H-bond and π–anion interaction), Arg442 (hydrophobic and π–cation interactions), and Asp69 (π–anion interaction) residues at the catalytic site (Figure 4d). In the allosteric inhibition mode, the 5-, 7-, 4′-, and 6′-OH groups of oxyresveratrol (**5**) formed six H-bonds with the Thr274, Thr290, Cys342, Ile272, Asn259, and Glu296 residues, showing −6.98 kcal/mol binding energy. In addition, oxyresveratrol (**5**) displayed hydrophobic interactions with Ala292, Trp15, and Ser291 residues (Figure 4j). The norartocarpetin (**6**)–*α*-glucosidase complex at the catalytic site showed a −6.64 kcal/mol binding energy, with an H-bond between the OH group and the Gln353 residue, and a hydrophobic interaction with the Val216 residue. In addition, the aromatic rings of norartocarpetin (**6**) showed a π–cation and two π–anion interactions with the Arg442, Glu277, and Asp352 residues, and also with acarbose (Figure 4e). In the allosteric inhibition mode, three OH- groups and one carbonyl group formed four H-bonds with Lys13, Lys16, Thr274, and Glu11. In addition, norartocarpetin (**6**) showed a hydrophobic interaction with the Ala292 and Lys13 residues, and its aromatic rings showed a π–anion interaction with the Glu271 residue (Figure 4k). Catalytic inhibition by kuwanon G (**7**) showed a −5.99 kcal/mol binding energy, forming six H-bonds between four OH groups and the Asn350, Gln353, Glu277, Asp352, Asp242, and Glu411 residues. In addition, kuwanon G (**7**) hydrophobically interacted with the Phe303 residue (Figure 4f). In comparison, four OH groups and one carbonyl of kuwanon G (**7**) formed seven H-bonds with the Ser298, Asn259, Ile272, Asp341, Thr290, Ala292, and Arg270 residues at the allosteric site. Kuwanon G (**7**) also exhibited a hydrophobic interaction with the Ala292, Trp15, His295, Ile262, Ile272, and Arg263 residues, as well as with BIP. Moreover, the aromatic rings of kuwanon G (**7**) showed one π–cation (Lys13) and one π–anion interaction (Glu271) (Figure 4l).

### 3.6. Molecular Docking Analysis for PTP1B Inhibition

**Figure 5:**
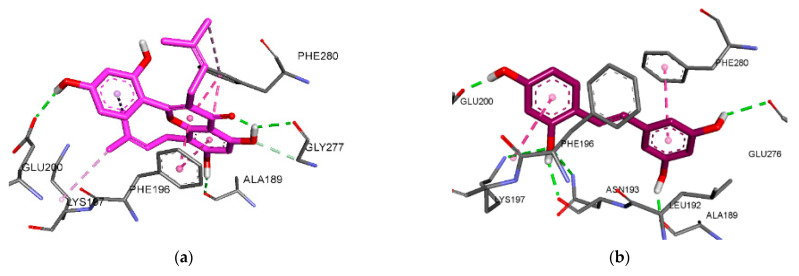

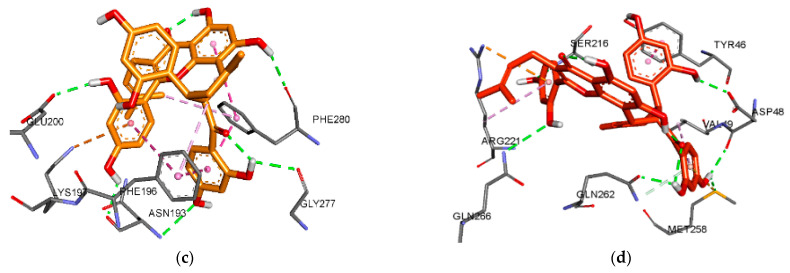
Molecular docking models for PTP1B inhibition at allosteric (**a**–**c**) and catalytic (**d**) sites by kuwanon C (**1**) (**a**), oxyresveratrol (**5**) (**b**), and kuwanon G (**7**) (**c**,**d**).

Molecular docking analyses with PTP1B were conducted for kuwanon C (**1**), oxyresveratrol (**5**), and kuwanon G (**7**). The ligand–enzyme complexes of the three test compounds, and of compound A and compound B, were rigidly placed in the same pocket as PTP1B using AutoDock 4.2. The binding energies of the test compounds, along with their H-bond, hydrophobic, and electrostatic interactions (shown by the green, pink, and orange lines, respectively), are listed in Table 4, Figure 5 and Figure S2.

In allosteric inhibition mode, three H-bonds formed between the three OH groups of kuwanon C (**1**) and the Gln200, Gly277, and Ala189 residues. Additionally, kuwanon C (**1**) showed the same hydrophobic interacting residues, Phe196, Phe280, Lys197, and Leu192, as compound B, the allosteric inhibitor (Figure 5a). Similarly, oxyresveratrol (**5**) displayed a hydrophobic interaction with Phe280, Phe196, and Leu192 residues, and formed six H-bonds between its four OH groups and the Asn193, Lys197, Glu200, Glu276, and Ala189 residues (−6.98 kcal/mol) (Figure 5b). On the other hand, kuwanon G (**7**) interacted with both the catalytic site and the allosteric site of PTP1B, indicating it as a mixed inhibitor. The kuwanon G (**7**)–PTP1B complex at the allosteric site exhibited five H-bonds with the Asn193, Gly277, Phe280, and Glu200 residues (−7.11 kcal/mol). Kuwanon G (**7**) also showed the same hydrophobic interacting residues as compound B—Phe196, Phe280, Ile281, and Leu192—and showed a π–cation interaction with the Lys197 residue (Figure 5c). In the catalytic inhibition mode, seven H-bonds formed between the six OH groups of kuwanon G (**7**) and the Ser216, Gln266, Asp48, Met258, Gln262, and Tyr46 residues, similarly to compound A, a catalytic inhibitor. The aromatic rings of kuwanon G (**7**) showed a π–cation interaction with the Arg221 residue (Figure 5d).

**Table 4:** Binding site residues and docking scores for compounds and known inhibitors of PTP1B (PDB: 1NNY and 1T49) obtained using AutoDock 4.2.

Compounds	Binding Energy ^1^	Number of H-Bonds	H-Bond Interacting Residues	Hydrophobic Interacting Residues	Electrostatic Interacting Residues
Kuwanon C (**1**)	−7.54	**3**	Glu200, Gly277, Ala189	Phe196(Pi-Pi Stacked), Phe280(Pi-Pi Stacked, Pi-Alkyl), Lys197(Alkyl), Leu192(Pi-Alkyl)	
Oxyresveratrol (**5**)	−6.98	**6**	Asn193, Lys197, GLu200, Glu276, Ala189	Phe280(Pi-Pi Stacked), Phe196(Amide-Pi Stacked), Leu192(Pi-Alkyl)	
Kuwanon G (**7**)	−6.26	**7**	Ser216, Gln266, Asp48, Met258, Gln262, Tyr46	Tyr46(Pi-Pi Stacked), Ala217(Alkyl), Ile219(Alkyl), Trp179(Pi-Alkyl), Val49(Pi-Alkyl), Arg221(Pi-Alkyl)	Arg221(Pi-Cation)
	−7.11	**5**	Asn193, Gly277, Phe280, Glu200	Phe196(Pi-Pi Stacked, Pi-Pi T-shaped, Pi-Alkyl), Phe280(Pi-Pi Stacked, Pi-Pi T-shaped, Pi-Alkyl), Ile281(Alkyl), Leu192(Pi-Alkyl)	Lys197(Pi-Cation)
Compound A ^2^	−10.2	**10**	Ser216, Ala217, Gly218, Ile219, Gly220,Arg221, Arg254, Asp48	Ala217(Pi-Sigma, Pi-Alkyl), Tyr46(Pi-Sigma, Pi-Pi Stacked), Ala27(Pi-Pi Stacked)	
Compound B ^3^	−9.08	**2**	Asn193, Glu276	Phe196(Pi-Sigma, Pi-Alkyl), Phe280(Pi-Pi Stacked, Pi-Pi T-shaped, Pi-Alkyl), Ile281(Alkyl), Leu192(Pi-Alkyl), Ala189(Pi-Alkyl)	

^1^ Estimated binding energy of the ligand–receptor complex (kcal/mol). ^2^ 3-({5-[*N*-acetyl-3-{4-[(carboxycarbonyl)(2-carboxyphenyl)amino]-1-naphthyl}-l-alanyl)amino]pentyl}oxy)-2-naphthoic acid, a known catalytic inhibitor. ^3^ 3-(3,5-dibromo-4-hydroxy-benzoyl)-2-ethyl-benzofuran-6-sulfonic acid (4-sulfamoyl-phenyl)-amide, a known allosteric inhibitor.

## 4. Discussion

Many natural products, characterized by enormous structural and chemical diversity, show potent bioactivity, with lower cytotoxicity and side effects than synthetic drugs [40]. New therapeutic agents derived from natural products are needed to treat DM and its complications with fewer side effects than are caused by currently available medications. We selected *Morus alba* branches for the in vitro assay, as well as the kinetic study and molecular docking analysis, of two enzymes involved in DM and its complications. All test compounds showed stronger inhibitory activity against α-glucosidase than the positive control, especially oxyresveratrol (**5**) and kuwanon G (**7**). Among them, kuwanon C (**1**), which possesses a prenyl moiety, showed a more potent α-glucosidase inhibitory activity than dihydromorin (**3**) and norartocarpetin (**6**). A similar result was reported in a recent study, which found that the presence of a prenyl moiety enhanced the α-glucosidase inhibitory activity. Similarly, the prenylation of C-8 can enhance the hydrophobic interactions between compounds and α-glucosidase, leading to an increase in inhibitory activity [41]. On the other hand, dihydromorin (**3**) and norartocarpetin (**6**) showed similar IC_50_ values, but the difference between the structures was only a C2–C3 double bond or an OH group on C-3. Tadera et al. [42] reported that increases in the number of OH groups and the presence of OH groups on C-3 and C-4′ in flavonoids were important for α-glucosidase inhibitory activity. Moreover, a C2–C3 double bond enhances α-glucosidase inhibition activity by increasing the π conjugation of the linkage in rings B and C, inducing near-planarity in the two rings. Thus, compounds with a near-planar structure can easily interact with the hydrophobic residue, which increases the α-glucosidase inhibitory activity [7]. Similarly, oxyresveratrol (**5**) showed the most potent activity against PTP1B, with an IC_50_ value of 2.85 μM, followed by kuwanon G (**7**), kuwanon C (**1**), dihydromorin (**3**), and moracin M (**2**). Our data are similar to those reported in previous studies, except for norartocarpetin (**6**), and we are the first to report the IC_50_ value of dihydromorin (**3**) (180.22 μM). Zhao et al. [43] reported that kuwanon C (**1**) previously showed higher inhibitory activity than norartocarpetin (**6**) at the same tested concentration (58% inhibition for kuwanon C (**1**) and 20% inhibition for norartocarpetin (**6**) at 20 μg/mL). Contrary to our results, norartocarpetin (**6**) exhibited potent activity against PTP1B (5.18 μM vs. >100 μg/mL) [44], possibly due to differences in the conditions of the PTP1B inhibition experiment. The presence of nonpolar and hydrophobic moieties enhanced the PTP1B inhibitory activity as well as cell permeability [45,46]. Thus, the inhibitory activities of oxyresveratrol (**5**), kuwanon G (**7**), and kuwanon C (**1**) were likely caused by an increase in bioavailability due to the presence of a prenyl group and multiple aromatic rings.

Although most of the compounds tested here have been subjects of previous reports on the inhibition of α-glucosidase and PTP1B, no studies have examined enzymes’ kinetics, or performed molecular docking simulations, except on oxyresveratrol (**5**) and kuwanon G (**7**). We conducted enzyme kinetic studies of the enzymes and compounds **1**–**3** and **5**–**7**. All compounds were mixed-type inhibitors of α-glucosidase, binding to the catalytic and allosteric sites of the free enzyme and enzyme–substrate complex. Kuwanon G (**7**) was also a mixed-type inhibitor of PTP1B, as was found in the results of Paudel et al. [47]. In contrast, kuwanon C (**1**) and oxyresveratrol (**5**) were non-competitive inhibitors of PTP1B. The *K*_i_ value defines the affinity between an enzyme and its inhibitors, i.e., lower *K*_i_ values indicate a better affinity between enzyme and inhibitor. Oxyresveratrol (**5**) showed the lowest *K*_i_ value (1.14 μM) against α-glucosidase, which implied that it had the strongest affinity with the enzyme, followed by kuwanon G (**7**), moracin M (**2**), kuwanon C (**1**), dihydromorin (**3**), and norartocarpetin (**6**). Similarly, the *K*_i_ value of oxyresveratrol (**5**) against PTP1B was the lowest (2.16 μM), followed by those of kuwanon G (**7**) and kuwanon C (**1**).

Based on the results of our enzyme kinetic study, we conducted molecular docking analyses of compounds **1**–**3** and **5**–**7** using isomaltase from baker’s yeast (71% identity and 84% similarity for sequence alignment) and its catalytic ligand α-d-glucose (PDB ID: 3A4A), because the structure of α-glucosidase derived from *Saccharomyces cerevisiae* is not crystallized [48]. Isomaltase contains four highly conserved regions: Ⅰ (residues 107–112), Ⅱ (residues 210–218), Ⅲ (residues 277–280), and Ⅳ (residues 347–352) [49]. The three conserved residues, Asp (region Ⅱ), Glu (region Ⅲ), and Asp (region Ⅳ), and His (regions Ⅰ and Ⅳ) are important catalytic residues in isomaltase and α-glucosidase [48]. The results for α-glucosidase docking show that compounds **1**–**3** and **5**–**7** interacted with the residues similarly to those used by acarbose, a known competitive inhibitor, as well as its surrounding residues. On the other hand, compounds **1**–**3** and **5**–**7** became bound to a different pocket in the catalytic site, but interacted with residues similarly to those used by BIP, a known non-competitive inhibitor. A recent study reported four allosteric sites for α-glucosidase (sites 1–4) [50], and we found that compounds **1**–**3** and **5**–**7** and BIP showed allosteric inhibition by interacting with site 4. Moreover, kuwanon C (**1**), dihydromorin (**3**), norartocarpetin (**6**), and kuwanon G (**7**) showed lower binding energies for allosteric inhibition than catalytic inhibition, indicating that these compounds preferentially bind to allosteric sites. On the other hand, moracin M (**2**) and oxyresveratrol (**5**) showed lower binding energies for catalytic inhibition than allosteric inhibition, indicating that these compounds preferentially bind to catalytic sites.

We used the results of our enzyme kinetic study to conduct a molecular docking analysis of kuwanon C (**1**), oxyresveratrol (**5**), and kuwanon G (**7**), using the complex structures of PTP1B, along with compound A (PDB ID: 1NNY) and compound B (PDB ID: 1T49). Kuwanon C (**1**) and oxyresveratrol (**5**) became bound to allosteric sites, whereas kuwanon G (**7**) bound to both catalytic and allosteric sites, although it had a lower binding energy for allosteric inhibition than catalytic inhibition, indicating its preference for allosteric sites. In addition, the results of the PTP1B docking analysis show that kuwanon C (**1**), oxyresveratrol (**5**), and kuwanon G (**7**) interacted with residues similarly to those used by compound B, a known non-competitive inhibitor. The allosteric site is found on the C-terminal portion of PTP1B, and on the side with α3 (residues 186–200), α6 (264–281), and α7 helices (287–295). Some residues of α3, α6, α7, and loop 11 are involved in the regulation of PTP1B function [51,52]. Kuwanon C (**1**), oxyresveratrol (**5**), kuwanon G (**7**), and compound B exhibited interactions with residues of the *α*3 and *α*6 helices. The residues interacting with kuwanon C (**1**), oxyresveratrol (**5**), kuwanon G (**7**), and compound B were Glu200, Ala189, Phe196, Lys197, Leu192, and Asn193 on the α3 helix; and Gly277, Phe280, Ile281, and Glu276 on the α6 helix. Therefore, those compounds were identified as non-competitive inhibitors. On the other hand, kuwanon G (**7**) also exhibited interactions with residues similarly to those used by compound A. The base of the active site is formed by residues from His214 to Arg221, which belong to the *β*12 sheet, loop 15, and the *α*4 helix. This site is surrounded by loop 1, loop 4, loop 13 (WPD loop), and loop 17 [53]. The residues interacting with kuwanon G (**7**) and compound A were Ser215, Ala217, and Gly218 (loop15); Ile219, Arg221, and Gly220 (α4 helix); Asp48, Tyr46, and Val49 (loop 1); Trp179 (WPD loop); and Met258 and Gln262 (loop 17). Among the interactions displayed by kuwanon G (**7**), the Asp48 and Tyr46 residues of the α4 helix were related to the tyrosine moiety of the substrate. Additionally, the WPD loop, including residues 177–185, is essential for catalysis into a substrate, after which it converts to its closed state. However, this state can be prevented by the allosteric inhibitor in the α3-α6-α7 helices [52]. The connection between the whole WPD loop and parts of the α3 and α7 helices is weakened by an allosteric inhibitor, causing it to maintain its open state and reducing catalytic activity [51]. Kuwanon G (**7**) likely interrupts the binding between the substrate and the catalytic site by interacting with residues from the α3 and α6 helices. Therefore, our results indicate that kuwanon G (**7**) interacted with both catalytic and allosteric sites as a mixed-type inhibitor.

In the two antioxidant mechanism-related assays, the AGE formation inhibitory and ONOO^−^ scavenging assays, most of the test compounds showed stronger activities than the positive controls, aminoguanidine and l-penicillamine, respectively. The results of the AGE formation inhibitory activity assay were similar to those for ONOO^−^ scavenging activity. In this study, the IC_50_ value (77.29 μM) required to inhibit AGE formation using norartocarpetin (**6**), and the IC_50_ values (12.92, 2.26, 2.37, 3.01, and 6.35 μM, respectively) required to inhibit ONOO^−^ using kuwanon C (**1**), dihydromorin (**3**), oxyresveratrol (**5**), norartocarpetin (**6**), and kuwanon G (**7**), have been reported for the first time. In the present study, kuwanon C (**1**) showed moderate activity in the ONOO^−^ scavenging assay, while its activity in the AGE formation inhibition assay could not be determined at the tested concentration up to 100 μg/mL. On the other hand, moracin M (**2**) exhibited the most potent bioactivity against both AGE formation, with an IC_50_ value of 7.15 μM, and ONOO^−^, with an IC_50_ value of 2.42 μM, which are similar to the results of a previous study [54]. The presence of an OH group on position 3 of ring C, as well as a catechol moiety on ring B and a C2–C3 double bond, enhances ONOO^−^ scavenging activity in flavonoids. The 3-OH group on ring C was positively influenced by a substituent 5- or 7-OH. Moreover, the 4-keto group can weaken the intramolecular H-bond, resulting in the optimal reactivity of the 3-OH group. The monophenol structure showed less activity than resorcinol [55]. The strong activity of dihydromorin (**3**) could be due to the presence of 3-OH and a resorcinol group. Furthermore, the IC_50_ values of flavones (i.e., luteolin) and flavonols (i.e., taxifolin) do not show significant differences [56], with results similar to those derived for dihydromorin (**3**) and norartocarpetin (**6**), which also showed no significant difference in IC_50_ value. On the other hand, the difference between kuwanon C (**1**) and norartocarpetin (**6**) comprises the presence of a prenyl moiety, and norartocarpetin (**6**) showed more potent activity than kuwanon C (**1**), indicating that the OH group could be more influential in ONOO^−^ scavenging than the prenyl moiety. This trend is supported by research suggesting that prenylation does not significantly affect the inhibition of lipid peroxidation by ONOO^−^ [57]. Since the results regarding the ONOO^−^ scavenging activity of flavonoids **1**, **3**, and **6** mirrored those derived for AGE formation inhibitory activity, the effect of the relationship between structure and inhibitory activity on AGE formation can be similarly explained.

Overall, our results illustrate that the compounds isolated from *Morus alba* branches could be used as significant therapeutic or preventive agents for DM and its complications through their antioxidant activity. However, further studies should be conducted to characterize the relationship of ONOO^−^ with two DM-associated enzymes, and the main mechanism, using an insulin-resistant cell line or a DM animal model.

## 5. Conclusions

Our results demonstrate that *Morus alba* branch extracts and their components exhibit anti-diabetic activity, including α-glucosidase and PTP1B inhibition. We also found inhibitory activity against AGE formation and ONOO^−^. All tested compounds showed better inhibitory effects against α-glucosidase than acarbose, with IC_50_ values ranging from 1.44 to 47.35 μM; moreover, oxyresveratrol (**5**) and kuwanon G (**7**) showed more potent inhibitory effects against PTP1B. The enzyme kinetic study indicated that all compounds showed a mixed-type inhibition pattern toward α-glucosidase. While kuwanon G (**7**) exhibited a mixed-type inhibition mode toward PTP1B, kuwanon C (**1**) and oxyresveratrol (**5**) showed a non-competitive inhibition pattern. These results are further supported by our molecular docking analysis, which showed that all compounds interacted with similar residues known to be important in catalytic and allosteric sites. Additionally, most of the compounds, especially moracin M (**2**), exhibited potent ONOO^−^ scavenging activity and AGE formation inhibition. Thus, proving the inhibition mechanism via computational and experimental studies could be useful in the development of functional foods to treat DM and its complications.

## Figures and Tables

**Table 1:** *α*-glucosidase, PTP1B, AGEs, and peroxynitrite inhibitory activities of MeOH extract of Morus alba branch and its fractions.

Fractions	IC_50_ Values (μg/mL) ^1^			
	α-Glucosidase	PTP1B	AGEs	ONOO^−^
MeOH ext.	100.3 ± 0.55	55.29 ± 27.46	23.06 ± 0.84	12.01 ± 1.43
CH_2_Cl_2_ fr.	135.3 ± 0.69	12.86 ± 2.45	48.64 ± 6.38	14.63 ± 0.92
EtOAc fr.	2.74 ± 0.15	8.09 ± 0.08	6.40 ± 0.31	6.74 ± 0.15
*n*-BuOH fr.	241.7 ± 0.10	15.05 ± 1.57	36.65 ± 2.00	10.98 ± 0.46
H_2_O fr.	457.9 ± 0.98	>100	>100	22.11 ± 3.56
Acarbose ^2^	494.1 ± 1.19	-	-	-
Ursolic acid ^2^	-	4.56 ± 0.22	-	-
Aminoguanidine ^2^	-	-	52.62 ± 6.70	-
l-Penicillamine ^2^	-	-	-	1.37 ± 0.19

^1^ The 50% inhibition concentration (IC_50_) is expressed as the mean ± SD of triplicate experiments. ^2^ Positive controls used in the assays.

**Table 3:** Binding site residues and docking scores of compounds and known inhibitors of α-glucosidase (PDB: 3A4A), obtained using AutoDock 4.2.

Compounds	Binding Energy ^1^	Number of H-Bonds	H-Bond Interacting Residues	Hydrophobic Interacting Residues	Electrostatic Interacting Residues
Kuwanon C (**1**)	−6.66	**4**	Gln279, Arg315, Arg442, Asp307	Tyr158 (Pi-Pi T-shaped, Pi-Alkyl), Lys156 (Alkyl), Phe303 (Pi-Alkyl), Arg315 (Pi-Alkyl)	
	−8.47	**5**	Glu296, Ser298, Leu297, Glu271, Arg270	Ala292 (Alkyl), Lys13 (Alkyl), Ile263 (Alkyl), Ile272 (Alkyl), Ile262 (Alkyl), Arg263 (Alkyl)	Glu271 (Pi-Anion)
Moracin M (**2**)	−7.73	**5**	Arg442, Asp69, Gln182, Asp215, Glu411	Tyr72 (Pi-Pi T-shaped), Tyr158 (Pi-Pi T-shaped)	Arg442 (Pi-Cation)
	−7.48	**4**	Lys16, His295, Asn259, Thr274	Trp15 (Pi-Sigma, Pi-Pi T-shaped), Ala292 (Pi-Sigma)	
Dihydromorin (**3**)	−6.52	**6**	Gln279, Arg315, Arg442, Asp69, Glu277, Asp352	Tyr72 (Pi-Pi T-shaped)	Arg442 (Pi-Cation), Asp352 (Pi-Anion), Glu411 (Pi-Anion)
	−6.93	**4**	Glu296, Asn259, Glu271, Ser291	Ala292 (Pi-Sigma), Arg263 (Pi-Alkyl)	
Oxyresveratrol (**5**)	−7.72	**4**	Asp352, Asp215, Gln353, Glu411	Tyr72 (Pi-Pi T-shaped), Phe178 (Pi-Pi T-shaped), Val216 (Pi-Alkyl), Arg442 (Pi-Alkyl)	Arg442 (Pi-Cation), Asp69 (Pi-Anion), Glu277 (Pi-Anion), Asp352 (Pi-Anion)
	−6.98	**6**	Thr274, Thr290, Cys342, Ile272, Asn259, Glu296	Ala292 (Pi-Sigma), Trp15 (Pi-Pi T-shaped), Ser291 (Amide-Pi Stacked)	
Norartocarpetin (**6**)	−6.64	**1**	Gln353	Val216 (PI-Alkyl)	Arg442 (Pi-Cation), Glu277 (Pi-Anion), Asp352 (Pi-Anion)
	−7.39	**4**	Lys13, Lys16, Thr274, Glu11	Ala292 (Pi-Sigma), Lys13 (Pi-Alkyl)	Glu271 (Pi-Anion)
Kuwanon G (**7**)	−5.99	**6**	Asn350, Gln353, Glu277, Asp352, Asp242, Glu411	Phe303 (Pi-Pi Stacked)	
	−8.89	**5**	Ser298, Asn259, Ile272, Asp341, Thr290, Ala292, Arg270	Ala292 (Pi-sigma, Pi-Alkyl, Alkyl), Trp15 (Pi-Pi T-shaped), His295 (Pi-Alkyl), Ile262 (Pi-Alkyl), Ile272 (Pi-Alkyl), Arg263 (Pi-Alkyl)	Lys13 (Pi-Cation), Glu271 (Pi-Anion)
Acarbose ^2^	−8.6	**6**	His112, Ser241, Arg442, Asp352, Asp242, Asp69	Tyr158 (Pi-Sigma), Phe303 (Pi-Alkyl)	
BIP ^3^	−6.03	**6**	Lys16	Ala292 (Pi-Sigma, Alkyl), Trp15 (Pi-Pi T-shaped, Pi-Alkyl), Lys13 (Alkyl)	

^1^ Estimated binding energy of the ligand–receptor complex (kcal/mol). ^2^ Known catalytic inhibitor. ^3^ (Z)-butylidenephthalide, known allosteric inhibitor.

## Data Availability

Data is contained within the article and Supplementary Materials.

## Supplementary Materials

The following supporting information can be downloaded at https://www.mdpi.com/article/10.3390/antiox11020383/s1, Table S1: ^1^H and ^13^C NMR chemical shifts of compounds **1**, **3**, **6**, and **8**, Table S2: ^1^H and ^13^C NMR chemical shifts of compounds **2**, **4**, **5**, and **7**, Scheme S1: The extraction and fractionation of the *Morus alba* branches, Scheme S2: The isolation of compounds from the EtOAc fraction of the *Morus alba* branches, Figure S1: Molecular docking analysis (2D diagram) for α-glucosidase inhibition—2D-binding diagram of α-glucosidase inhibition at catalytic (A–F) and allosteric sites (G–L) by compounds **1**–**3** and **5**–**7**, respectively. (A,G) for kuwanon C (**1**); (B,H) for moracin M (**2**); (C,I) for dihydromorin (**3**); (D,J) for oxyresveratrol (**5**); (E,K) for norartocarpetin (**6**); (F,L) for kuwanon G (**7**), Figure S2: Molecular docking analysis (2D diagram) for PTP1B inhibition—2D-binding diagram of PTP1B inhibition at catalytic (D) and allosteric sites (A–C) by kuwanon C (**1**) (A), oxyresveratrol (**5**) (B), and kuwanon G (**7**) (C,D).
