# Supplementary material for: Inhibition Mechanism of Components Isolated from Morus alba Branches on Diabetes and Diabetic Complications via Experimental and Molecular Docking Analyses
Source: Antioxidants (Basel). 2022 Feb 14;11(2):383. doi: 10.3390/antiox11020383 (PMC8869400; doi:10.3390/antiox11020383)
Supplement: Supplementary file 1 [file antioxidants-11-00383-s001.zip › antioxidants-1563050-supplementary.pdf]

Supplementary Materials: Inhibition Mechanism of Components Isolated from *Morus alba* Branches on Diabetes and Diabetic Complications via Experimental and Molecular Docking Analyses

Ryeong-Ha Kwon [1], Niha Thaku [1], Binod Timalsina [1], Se-Eun Park [2,3], Jae-Sue Choi [2,*] and Hyun-Ah Jung [1,*]

[1] Department of Food Science and Human Nutrition, Jeonbuk National University, Jeonju 54896, Korea;
haha8447@gmail.com (R.H.K.); neehathaku@gmail.com (N.T.);
binodtimalsina19@gmail.com (B.T.)
[2] Department of Food Science and Nutrition, Pukyong National University, Busan 48513, Korea; gogo1685@naver.com
[3] Department of Biomedical Science, Asan Medical Institute of Convergence Science and Technology,
Seoul 05505, Korea
* Correspondence: choijs@pknu.ac.kr (J.S.C.); jungha@jbnu.ac.kr (H.A.J.); Tel.: +82-51-629-7547 (J.S.C.);
+82-63-270-4882 (H.A.J.)

Table S1. ^{1}H and ^{13}C NMR chemical shifts of compounds **1**, **3**, **6**, and **8**

Table S2. ^{1}H and ^{13}C NMR chemical shifts of compounds **2**, **4**, **5**, and **7**

Scheme S1. The extraction and fractionation of the *Morus alba* branches

Scheme S2. The isolation of compounds from the EtOAc fraction of the *Morus alba* branches

Figure S1. Molecular docking analysis (2D diagram) for α-glucosidase inhibition

Figure S2. Molecular docking analysis (2D diagram) for PTP1B inhibition

Table S1. ^{1}H and ^{13}C NMR chemical shifts of compounds **1**, **3**, **6**, and **8**

No.	Kuwanon C (**1**) [1]		Dihydromorin (**3**) [2]		Norartocarpetin (**6**) [2]		Kaempferol 7-*O*-*β*-D-glucopyranoside (**8**) [3]	
	δ_H (*J* in Hz)	δ_C	δ_H (*J* in Hz)	δ_C	δ_H (*J* in Hz)	δ_C	δ_H (*J* in Hz)	δ_C
2		161.7	5.39 (1H, d, *J* = 12 Hz)	79.9		165.9		147.5
3		121.3	4.79 (1H, d, *J* = 12 Hz)	72.4	7.14 (1H, s)	108.2		136.0
4		184.0		198.9		184.3		176.1
4a		105.3		101.8		105.1		104.7
5		157.0		168.5		163.0	12.4 (OH, s)	160.3
6	6.24 (1H, s)	98.9	5.88 (1H, d, *J* = 1.2 Hz)	96.2	6.19 (1H, d, *J* = 2.5 Hz)	99.8	6.41 (1H, d, *J* = 1.5Hz)	98.8
7		163.6		164.9		164.1		162.7
8		107.5	5.92 (1H, d, *J* = 1.8 Hz)	97.1	6.42 (1H, d, *J* = 1.8 Hz)	94.8	6.79 (1H, d, *J* = 2.5Hz)	94.4
8a		162.6		165.2		163.3		155.7
1'		113.5		115.5		110.7		121.5
2'		157.8		158.5		159.4	8.06 (1H, d, *J* = 8.5Hz)	129.6
3'	6.44 (1H, d, *J* = 2.0 Hz)	103.7	6.37 (1H, d, *J* = 5.4 Hz)	103.6	6.42 (1H, d, *J* = 3.0 Hz)	104.1	6.93 (1H, d, *J* = 9.0Hz)	115.5
4'		160.6		160.1		160.4		159.4
5'	6.41 (1H, dd, *J* = 2.4, 8.4 Hz)	107.8	6.36 (1H, d, *J* = 1.2 Hz)	107.9	6.46 (1H, dd, *J* = 2.4, 8.5 Hz)	109.1	6.93 (1H, d, *J* = 9.0Hz)	115.5
6'	7.08 (1H, d, *J* = 8.4 Hz)	132.0	7.22 (1H, d, *J* = 9.0 Hz)	130.9	7.77 (1H, d, *J* = 8.5 Hz)	131.0	8.06 (1H, d, *J* = 8.5Hz)	129.6
1''	3.11 (2H, s)	24.8					5.05 (1H, d, *J* = 7.0Hz)	99.9
2''	5.10 (1H, t, *J* = 5.2 Hz)	122.9						73.1
3''		132.6						76.4
4''	1.40 (3H, s)	17.6						69.6
5''	1.58 (3H, s)	25.8						77.1
6''								60.6
1'''	3.36 (2H, s)	22.3						
2'''	5.17 (1H, t, *J* = 6.0 Hz)	123.4						
3'''		132.4						
4'''	1.56 (3H, s)	17.7						
5'''	1.60 (3H, s)	25.9						

[1] CD_3OD, ^{1}H (400 MHz), ^{13}C (100 MHz), [2] CD_3OD, ^{1}H (500 MHz), ^{13}C (125 MHz), [3] $(CD_3)_2SO$, ^{1}H (500 MHz), ^{13}C (125 MHz)

Table S2. ^{1}H and ^{13}C NMR chemical shifts of compounds **2**, **4**, **5**, and **7**

No.	Moracin M (**2**) [1]		*β*-Sitosterol glucoside (**4**) [2]		Oxyresveratrol (**5**) [3]		Kuwanon G (**7**) [4]	
	δ_H (*J* in Hz)	δ_C	δ_H (*J* in Hz)	δ_C	δ_H (*J* in Hz)	δ_C	δ_H (*J* in Hz)	δ_C
1				37.5	CD_3OD, 400 MHz	117.8		
2		156.1		30.3		157.3		162.5
3	6.92 (1H, s)	102.2		78.6	6.30 (1H, m)	108.3		121.7
3a		123.0						

4	7.35 (1H, d, *J* = 8.0 Hz)	122.0		40.0		159.2		183.9
4a								105.7
5	6.75 (1H, dd, *J* = 2.0, 8.0 Hz)	113.3		140.9	6.30 (1H, m)	103.5		157.8
6		156.8	5.34 (1H, d, *J* = 4.8 Hz)	121.9	7.32 (1H, d, *J* = 6.0 Hz)	128.4	5.94 (1H, s)	98.5
7	6.92 (1H, d, *J* = 1.5 Hz)	98.4		32.2				165.7
7a		157.2						
8				32.1				108.6
8a								162.5
9				50.3			3.19 (2H, br)	24.7
10				36.9			5.17 (1H, t, *J* = 7.2 Hz)	123.0
11				21.3				132.7
12				39.3			1.64 (3H, s)	25.9
13				42.5			1.46 (3H, s)	17.7
14				56.8			4.34 (1H, d, *J* = 8.4 Hz)	23.1
15				24.5			5.19 (1H, brs)	124.6
16				28.6				134.4
17				56.2			1.49 (3H, brs)	25.9
18			0.65 (3H, s)	12.0			1.95 (2H, br)	39.1
19			0.98 (3H, d, *J* = 6.6 Hz)	19.4			3.35 (1H, s)	23.1
20				36.4			4.58 (1H, brd, *J* = 33 Hz)	49.9
21			1.06 (3H, m)	19.0				210.2
22				34.2				115.9
23				26.4				165.9
24				46.0			5.94 (1H, s)	103.7
25				29.5				165.7
26			0.92 (3H, s)	19.2			5.90 (1H, d, *J* = 8.4 Hz)	108.6
27			0.86 (3H, t, *J* = 6.0 Hz)	20.0			7.34 (1H, brs) or 7.15 (1H, d, *J* = 8.4 Hz)	132.7
28				23.4				123.0
29			0.88 (3H, t, *J* = 6.0 Hz)	12.2				161.8
30							6.14 (1H, brs)	102.9
31								161.1
32							6.08 (1H, dd, *J* = 2.4, 8.4 Hz)	108.2
33							6.75 (1H, d, *J* = 7.2 Hz)	134.4
1'		133.8	5.04 (1H, d, *J* = 7.2 Hz)	102.6		142.2		113.8
2'	6.78 (1H, d, *J* = 2.0 Hz)	103.9		75.3	6.43 (1H, d, *J* = 2.4 Hz)	105.6		161.8
3'		159.9		78.5		159.6	6.47 (1H, brs)	103.6
4'	6.27 (1H, t, *J* = 2.5 Hz)	103.5		71.7	6.12 (1H, t, *J* = 2.4 Hz)	101.2		162.5
5'		159.9		78.1		159.6	6.50 (1H, s)	108.0

6'	6.78 (1H, d, *J* = 2.0 Hz)	103.9		62.8	6.43 (1H, d, *J* = 2.4 Hz)	105.6	7.34 (1H, brs) or 7.15 (1H, d, *J* = 8.4 Hz)	132.7
2''								
3''								
4''								
5''								
6''								
α					6.80 (1H, d, *J* = 16.4 Hz)	124.8		
β					7.26 (1H, d, *J* = 16 Hz)	126.5		

[1] CD_3OD, ^1H (500 MHz), ^{13}C (125 MHz), [2] C_5D_5N, ^1H (600 MHz), ^{13}C (150 MHz), [3] CD_3OD, ^1H (400 MHz), ^{13}C (100 MHz), [4] CD_3OD, ^1H (600 MHz), ^{13}C (150 MHz)

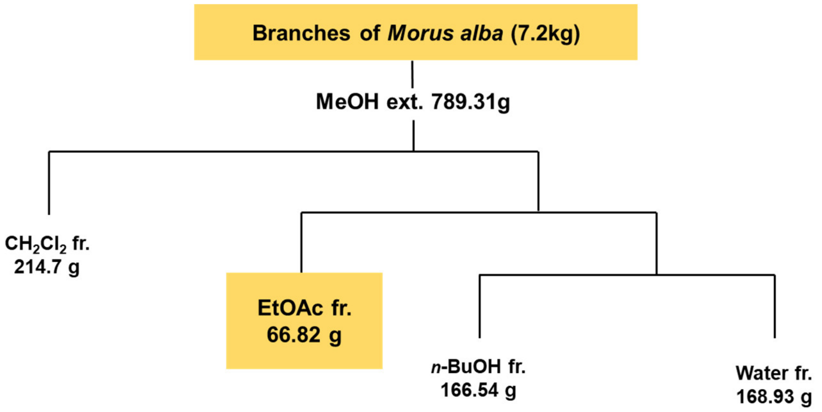

Scheme S1. The extraction and fractionation of the *Morus alba* branches

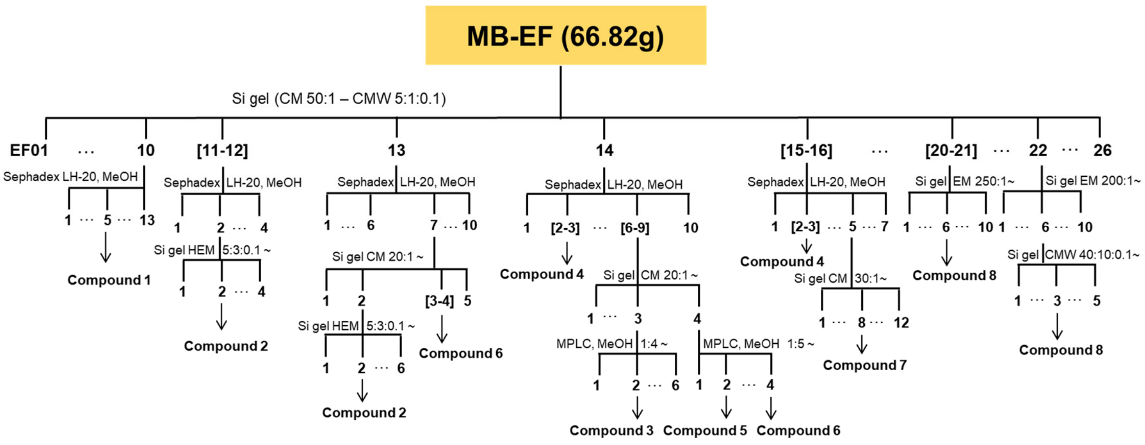

Scheme S2. The isolation of compounds from the EtOAc fraction of the *Morus alba* branches

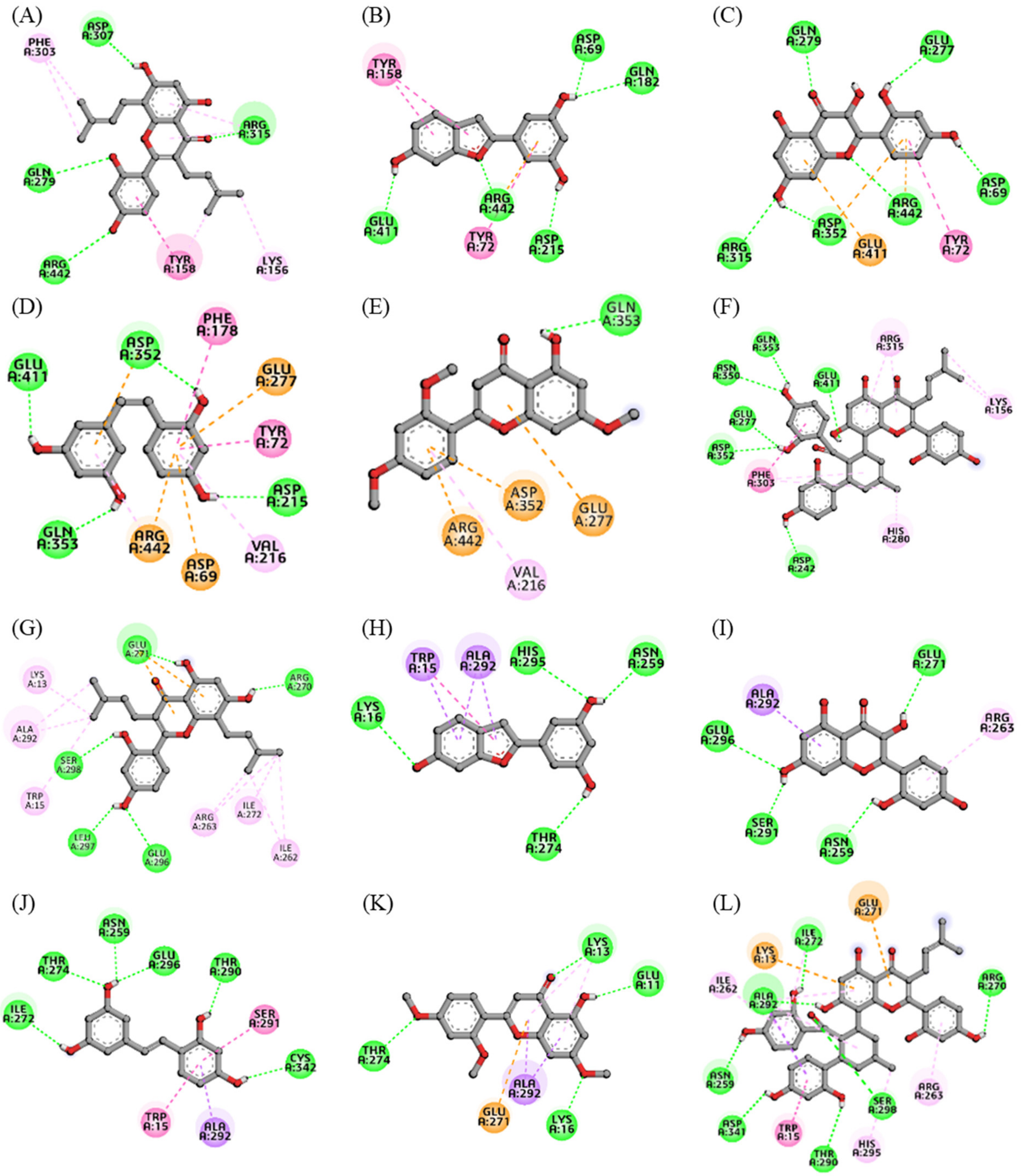

Figure S1. 2D-binding diagram of α-glucosidase inhibition at catalytic (A-F) and allosteric sites (G-L) by compounds **1**–**3** and **5**–**7**, respectively: A and G for kuwanon C (**1**); B and H for moracin M (**2**); C and I for dihydromorin (**3**); D and J for oxyresveratrol (**5**); E and K for norartocarpetin (**6**); F and L for kuwanon G (**7**)

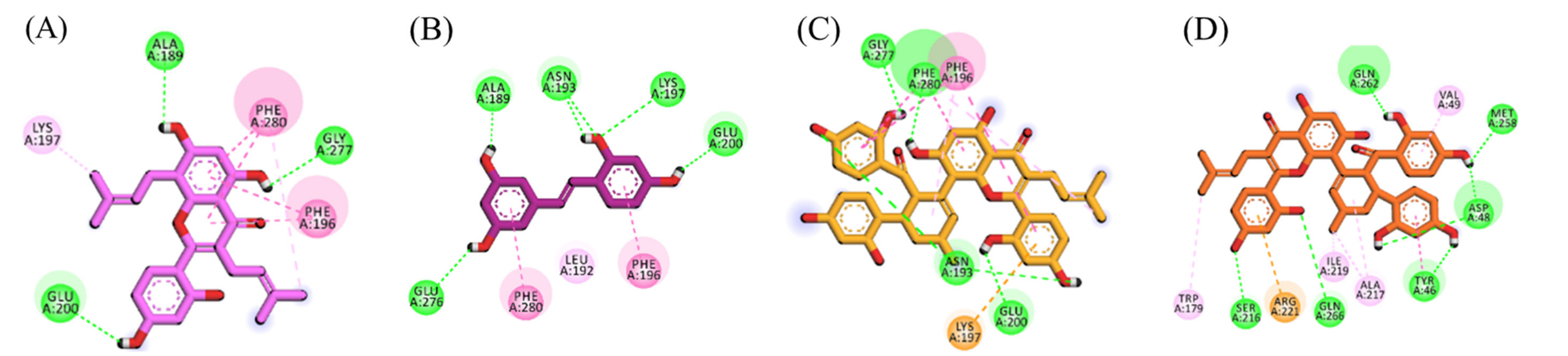

Figure S2. 2D-binding diagram of PTP1B inhibition at catalytic (D) and allosteric sites (A-C) by kuwanon C (**1**) (A), oxyresveratrol (**5**) (B), and kuwanon G (**7**) (C and D).
